# Irrational use of antibiotics in the Moshi Municipality Northern Tanzania: a cross sectional study

**DOI:** 10.11604/pamj.2018.31.165.15991

**Published:** 2018-11-08

**Authors:** Erick Alexander Mboya, Leah Anku Sanga, James Samwel Ngocho

**Affiliations:** 1 Kilimanjaro Christian Medical University College, P.O. Box 2240 Moshi, Tanzania; 2 Institute of Public Health, Kilimanjaro Christian Medical University College, P.O. Box 2240 Moshi, Tanzania

**Keywords:** Antibiotics, antimicrobials, antibiotic use, antibiotic resistance, irrational use of antibiotics

## Abstract

**Introduction:**

Irrational use of antibiotics includes prescription of incorrect doses, self-medication and treatment of non-bacterial illness. As a direct consequence of irrational antibiotic use, resistance to the commonly available antibiotics has been increasing rapidly. This phenomenon is associated with poorer health outcomes, longer hospitalization, increased cost to both the patient and government, and increased mortality. The aim of this study was to determine the prevalence of, and examine factors associated with, irrational use of antibiotics in the Moshi municipality, Northern Tanzania.

**Methods:**

We conducted a cross-sectional descriptive study between April and May 2017 in the Moshi municipality. Twelve drug outlets, of which five were pharmacies and seven accredited drug dispensing outlets (ADDOs), were selected at random. On exiting these outlets, all adults who had purchased antibiotics were interviewed using structured questionnaires.

**Results:**

A total of 152 adults were enrolled in this study. The median (QR) age was 31 years (25-42). The majority, 94 (61.8%), of the participants were female. ADDOs contributed 81 (53.3%) and pharmacies contributed 71 (46.7%) of all participants. Overall, 135 (88.8%) of antibiotic purchases were irrational. The most prevalent form of irrational antibiotic use was non-prescription usage; 116 of the 152 (76.3%) purchases fell in this category. Purchases of the incomplete dosage and purchases for non-bacterial illness were also widespread. Poor knowledge about the use of antibiotics had a significant association with irrational use of antibiotics (aOR=5.1, 95% CI: 1.80-15.15).

**Conclusion:**

Irrational use of antibiotics is highly prevalent in this population. Non-prescription use of antibiotics is the most prevalent form. Poor knowledge about antibiotic use plays a significant role in irrational antibiotic use. There is a need to review the accessibility of antibiotics in drug outlets.

## Introduction

Although antibacterial resistance is a natural phenomenon, human factors exacerbate its emergence and spread [1]. One of the major factors that greatly influences the development of resistance is the irrational use of antibiotics [2, 3]. Irrational use of antibiotics can take many forms, including the use of too many medicines per patient (polypharmacy), the inappropriate use of self-medication (often with prescription-only medicines), in non-bacterial infections, outside clinical guidelines, or with inadequate dosage or inappropriate route of administration such as overuse of injections when oral formulations would be more appropriate [2, 4]. All these factors expose bacteria to sub-optimal levels of antibiotics, which is not only therapeutically ineffective but also facilitates the formation of resistance against the drug by the bacteria [1]. Worldwide, irrational use of antibiotics is escalating, both in developed and developing countries [4]. It is estimated that 80 % of all antibiotics are used in the community, where prescribing and purchasing of antibiotics without prescription is common, especially in low- and middle-income countries where policies and regulations are often not implemented despite being in place [5,6]. Several studies have reported the prevalence of irrational use of antibiotics in developed countries. Here, irrational use of antibiotics is mainly due to excessive prescription by general practitioners influenced by diagnostic uncertainty [7]. Studies carried out in Southern and Eastern Europe recorded the prevalence of irrational use of antibiotics as approximately 30% and 19% respectively. The Southern region, included Greece, Italy, Malta and Spain, and the Eastern region included Lithuania, Poland and Romania [8-12]. Studies performed in Northern (Austria, Belgium, Czech Republic, Denmark, Ireland, Luxembourg, the Netherlands, Sweden and the UK) and Central (Croatia, Slovakia and Slovenia) European countries reported the lowest prevalence of irrational use of antibiotics, ranging from 3% in the Northern countries to 6% in the Central countries [10, 12, 13]. The prevalence of irrational antibiotic use in the Middle East varies significantly between countries, being as high as 39% in Jordan and Saudi Arabia, and as low as 4% in Israel [12]. In the African region, irrational use of antibiotics is largely in the form of non-prescription sale. Here, a significant number of people visit drug outlets before seeing a health worker when they fall ill [7, 14, 15]. Studies in Ethiopia and Zambia have shown that irrational use of antibiotics ranges from 75% to 100% [12, 16, 17] As a consequence of irrational antibiotic use, resistance to the commonly available antibiotics has been increasing rapidly. This phenomenon is associated with poorer health outcomes, longer hospitalization, increased cost to both the patient and government, and increased mortality [18, 19]. These effects are even more deleterious and pronounced in developing countries due to poor health systems, high incidence of infectious diseases, endemicity of HIV/AIDS and malnutrition [20]. In Tanzania, antibiotics are prescription-only medicines. They are sold in pharmacies and in accredited drug dispensing outlets (ADDOs), known locally as Duka la Dawa Muhimu (DLDM). Pharmacies are supervised by registered pharmacists, whereas in accredited outlets there are dispensers who have undergone a short training course. Despite regulations, the introduction of ADDOs, and improvements in dispensers' knowledge of treatment and dispensing practices in Tanzania, the irrational use of antibiotics remains at about 80% to 85% in retail pharmacies and other drug outlets [20-22]. It is almost impossible to reverse resistance to antibiotics once it is present in the pool of bacteria. However, through rational use of antibiotics, the spread and emergence of resistance can be slowed down before causing additional damage to the health and finances of the community [23]. Thus, the aims of this study are to determine the prevalence of the irrational use of antibiotics obtained from pharmacies and other drug outlets in the Moshi municipality, Northern Tanzania, and to examine the factors associated with the irrational use of antibiotics.

## Methods

A cross-sectional descriptive study was conducted from April 2017 to May 2017 in the Moshi municipality. The municipality is situated on the lowest slopes of Mount Kilimanjaro, a dormant volcano, which is the highest mountain in Africa and located in the Kilimanjaro region, Northern Tanzania. According to the census of 2012, the population of the Moshi municipality is 184,292 (National Bureau of Statistics, 2012). The Moshi municipality is divided into 11 wards. There are two main types of drug outlet in the area: pharmacies and ADDOs. In total, there are 20 pharmacies and about 37 ADDOs in the municipality. In the urban center of the municipality there are mainly pharmacies, whereas the outlying rural areas tend to be served by ADDOs. All drug outlets in the Moshi municipality that are recognized as pharmacies or ADDOs were included in the sampling. Sample size estimation was obtained based on the formula for precision.

$${\text{N}=\text{Z}}^{2}\text{p}\left(1-\text{p}\right){\text{/Σ}}^{2}$$

Prevalence was obtained from previous studies in the coastal regions of Morogoro and Pwani in Eastern Tanzania [21]. The minimum sample size required was 145. A total of five pharmacies and seven ADDOs were included in the study. All individuals buying antibiotics in the sampled outlets during the study period were included in the study if they met the inclusion criteria. Data were collected in the pharmacies and at the ADDOs immediately after a person purchased antibiotics. After consenting, an interview was conducted just outside the drug outlet using a structured questionnaire. Participants' demographic characteristics including age, sex, residence, education level, marital status and social-economic status were recorded. Social-economic status (SES) of participants was determined by their level of education, employment, type of housing and health-insurance status, and whether they had experienced any shortage of food in the preceding month. For each purchase, the antibiotic dispensed including the dosage was recorded. A check for a valid prescription was also made. If a prescription was held, the drugs dispensed were checked against the prescription. If the purchase matched the prescription (i.e. the correct antibiotic and dosage), it was classified as rational antibiotic use. No attempt was made to assess whether the prescribing of the doctor was correct. For antibiotics purchased without a prescription, the presenting illness or symptom leading to the purchase was obtained. In addition, the purchaser was asked if the dispenser suggested the treatment to them or whether they had requested the antibiotic because they or a friend had used it before for the treatment of a similar illness. If antibiotics were purchased without a prescription, or if the purchase did not match the prescription (i.e. wrong antibiotic or dosage), then the purchase was classified as irrational antibiotic use.

Knowledge about antibiotics was assessed using validated questions adapted from a WHO questionnaire used in a multi-country awareness survey [4]. Three of the questions in the tool were used to assess the participants' knowledge about antibiotics. These were: 1) when do you think you should you stop antibiotics once you've begun treatment' When you feel better or when you finish the dose as directed? 2) is it okay to use antibiotics given to someone else-for example a friend or a family member-as long they were used for treating the same illness? 3) If you are sick, is it okay to buy, or to request the same antibiotics from a doctor, if they helped you get better when you had same symptoms previously? Participants who correctly answered the three questions were considered to have good knowledge, while the remainder were considered to have poor knowledge. Data were entered in a database, cleaned, rechecked for completeness and analyzed using the Statistical Package for Social Science version 24 (IBM USA). The age profile of the study population was characterized by calculating the median age along with the interquartile range. Frequencies and percentages were used to summarize categorical variables, which included general characteristics of the study population (gender, age group, educational level, and marital, insurance and employment status), outlet type, antibiotics bought and presenting illness or symptoms. A univariate and multivariable logistic regression model was applied to determine the predictors of irrational use of antibiotics and odds ratios with their respective 95% confidence intervals (CI) were estimated. A p-value of less than 0.05 was considered to be statistically significant. Research was approved by the Kilimanjaro Christian Medical University College Research Ethics Committee. In addition, we obtained a permit from the municipal medical officer. Eligible clients gave written consent prior to enrollment in this study.

## Results

**Figure 1:**
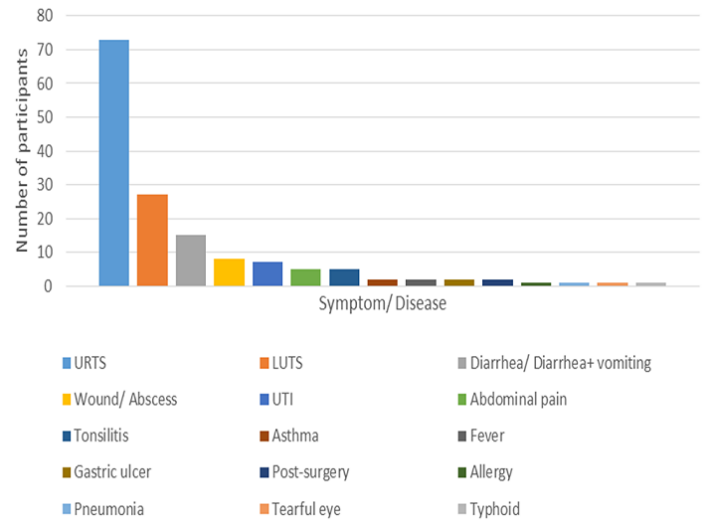
Common symptoms and diseases presented for buying antibiotics

A total of 152 adults were enrolled in this study and their median (IQR) age was 30.5 (25-42) years. The majority, 94 (61.8%), of participants were female. Most, 91 (59.9%), participants were married. More than half, 81 (53.3%), of participants made their purchases from the ADDOs (Table 1). The number of participants using antibiotics irrationally was assessed to be 135 (88.8%). The majority, 116 (76.3%), of purchases were without a prescription and 35 (23%) participants bought an incomplete dosage of less than 5 days. The most common antibiotics bought were Ampiclox, Amoxicillin, Metronidazole, Ciprofloxacin, Azithromycin and Erythromycin, with 41 (27%), 29 (18.4%), 14 (8.7%), 13 (8.1%), 10 (6.2%) and 10 (6.2%) purchases respectively (Table 2). Upper respiratory tract symptoms (URTS), such as cough and flu, were the most common presenting complaint and were present in 73 participants (48%). Lower urinary tract symptoms (LUTS) and diarrhea were the next most common symptoms and were present in 27 (17.8%) and 15 (9.9%) of the patients respectively (Figure 1). Significantly, participants with poor knowledge concerning how to use antibiotics had 5.5 higher odds of using antibiotics irrationally compared to those with good knowledge (cOR=5.5, 95% CI; 1.91-15.63, P value=0.002). Although not significant, participants who did not have health insurance had two times higher odds of using antibiotics irrationally compared to those with health insurance (cOR=2, 95% CI; 0.71-5.66, P value= 0.191). Other factors, such as the gender, age, educational level, employment status and marital status of participants, and the outlet type, were not significantly associated with irrational use of antibiotics (Table 3). In multivariate analysis, knowledge about antibiotic use was an independent predictor of irrational use of antibiotics when adjusted for formal employment and health insurance status. Participants with poor knowledge concerning the use of antibiotics had 5.1 higher odds of using antibiotics irrationally than those with good knowledge (aOR=5.1, 95% CI; 1.7-15.15). Although not significant when adjusted for formal employment status and knowledge concerning antibiotic use, participants without health insurance had 1.5 higher odds of using antibiotics irrationally compared to those with health insurance (aOR=1.5, 95% CI; 0.40 -5.04) (Table 3).

**Table 1:** General characteristics of the study population (n=152)

Variable	n	%
**Social demographic characteristics of participants**		
**Gender**		
Male	58	38.2
Female	94	61.8
**Age (years)**		
18-25	42	27.6
26-35	59	38.8
36-45	24	15.8
46-55	12	7.9
> 55	15	9.9
**Educational Level**		
Primary education	44	28.9
Secondary education	73	48
Diploma/Certificate	19	12.5
Bachelor Degree/Masters	16	10.6
**Formal employment**		
Yes	23	15.1
No	129	84.9
**Health Insurance**		
Yes	42	27.6
No	110	72.4
**Marital status**		
Married	91	59.9
Not married	61	40.1
**Outlet characteristics**		
**Outlet type**		
ADDOs	81	53.3
Pharmacy	71	46.7

**Table 2:** Ccommonly bought antibiotics (n=161)

Name of the antibiotic	n	%
Ampiclox tabs/syrup	41	25.5
Amoxicillin tabs/ syrup	29	18.0
Metronidazole 200mg	14	8.7
Ciprofloxacin 500mg	13	8.1
Azithromycin tabs	10	6.2
Erythromycin tabs/ syrup	10	6.2
Cephalexin 250mg	8	5.0
Cotrimoxazole/ Septrin tabs/ syrup	8	5.0
Amoxyclav tabs/ Augmentin syrup	7	4.3
Pen V	5	3.1
Ampicillin tabs/ syrup	3	1.9
Others	13	7.8

**Table 3:** factors associated with the irrational use of antibiotics

Variable	Total	Irrational use n (%)	Unadjusted OR (95% CI)	P-value	Adjusted OR (95% CI)	P-value
**Gender**						
Male	58	51(87.9)				
Female	94	84(89.4)	1.2 (0.41-3.21)	0.786		
**Age**						
35 and below	101	90 (89.1)	1.1 (0.38-3.14)	0.872		
Above 35	51	45 (88.2)				
**Education level**						
Primary education	44	38 (86.4)				
Secondary & above	108	97 (89.8)	1.4 (0.48-4.03)	0.540		
**Formal Employment**						
Yes	23	19(82.6)				
No	129	116(89.9)	1.9 (0.55-6.37)	0.312	1.0 (0.23-4.11)	0.960
**Health Insurance**						
Yes	42	35(83.3)				
No	110	100(90.9)	2.0 (0.71-5.66)	0.191	1.5 (0.40-5.04)	0.517
**Marital status**						
Married	91	81(89.0)				
Not married	61	54(88.5)	1.0 (0.34-2.66)	0.926		
**Outlet type**						
ADDOs	81	73(90.1)	1.3 (0.48-3.64)	0.586		
Pharmacy	71	62(87.3)				
**Knowledge on use of antibiotics**						
Good	38	28 (73.7)				
Poor	114	107(93.9)	5.5 (1.91-15.63)	0.002	5.1 (1.80-15.15)	0.003

Adjusted for Formal employment, health insurance and knowledge on use of antibiotics

## Discussion

The results of this study show that the irrational use of antibiotics in the Moshi municipality is 88.8%, with non-prescription usage being the most prevalent form. Other forms include incomplete dosage and using antibiotics to treat non-bacterial illnesses. Knowledge about the use of antibiotics appears to be the most important factor driving irrational use of antibiotics. Despite regulations for prescription-only drugs, including antibiotics, acquisition of antibiotics without prescription in the community was found to be prevalent in this study, accounting for 76.3% of antibiotics dispensed in both pharmacies and ADDOs. The policies and regulations in Tanzania requiring antibiotics to be sold only on prescription are not enforced and antibiotics are easily accessible through drug outlets. This may be driven by the desire of drug outlets to maximize profits, and by poor knowledge and enforcement of relevant policies and regulations. Similar results concerning the magnitude of non-prescription sale of antibiotics in Tanzania have been reported by Viberg et al, who found that 68% of antibiotics dispensed by private pharmacies across eight other districts of the country had no prescription. Easy accessibility of antibiotics without prescription from pharmacies and other drug outlets seems to be a common practice not only in Tanzania but also in other African nations and other developing countries, where dispensers rarely ask for a prescription and most antibiotics are given on request of the patient or at the suggestion of the dispenser [12]. Findings similar to this study have been reported from investigations using client-simulation methods in the Morogoro region of Tanzania, in Ethiopia and in India, with respective rates of non-prescription antibiotic use of 79%, 76% and 67% detected [16, 21, 24, 25]. In Northern and Western Europe self-medication with antibiotics is as low as 1%; this is due to enforcement of regulations with no easy accessibility of antibiotics from drug outlets [10].

Most antibiotics dispensed to participants in this study were to treat symptoms such as upper respiratory tract symptoms (48%)-like cough and flu-lower urinary tract symptoms (17.8%) and diarrhea (9.9%). URTS and diarrhea most often result from viral rather than bacterial infection. Hence, use of antibiotics in such cases is not typically indicated and serves to exacerbate the emergence and spread of resistance. It is noted that two other studies conducted in Tanzania found very similar levels of antibiotic prescribing for URTS to that reported here, with 47-48% of antibiotics dispensed to treat coughs and flu, compared to 48% in this study [21, 24]. Respiratory tract symptoms, diarrhea and wounds have been reported to be the major symptoms to be treated with antibiotics in drug outlets in Ethiopia, Vietnam, China, India and Europe [10, 16, 25-27]. The most common irrationally used antibiotic was Ampiclox, comprising over a quarter of all antibiotics bought irrationally. It is perhaps not surprising, therefore, that almost all susceptibility studies conducted in the region have found Ampicillin to be the least effective penicillin with resistance ranging from 85% to 98% [18, 20]. There is a similar picture for Amoxicillin, which is the second most irrationally used antibiotic in this study. More generally, Penicillins have been identified as the least effective antibiotics in the region [18-20]. This finding is in keeping with this study in which over half of all irrationally used antibiotics were Penicillins. Macrolides were the second most irrationally used antibiotics. These classes of antibiotics can be afforded by most people and, because of poor knowledge and their easy availability, they are commonly used to treat non-bacterial infections. In this study, an incomplete course of antibiotics (a supply of less than five days) was purchased by 23% of the participants. The reported incidence of incomplete dosage was much higher in another study performed in Morogoro region and in Kibaha, with 85% of dispensers willing to dispense an incomplete course of antibiotic. This difference can be explained by the differing methods used in the two studies: the second study used a ‘mystery shopper’ methodology, which can exaggerate the real magnitude of the problem [21]. Poverty may be a significant factor for many people purchasing an incomplete course of medication. The willingness of the dispenser to provide antibiotics without prescription, in incomplete dosage or to treat non-bacterial symptoms and illnesses facilitates the easy accessibility and the irrational use of antibiotics with the resulting negative consequences.

In this study knowledge about the use of antibiotics was highly associated with their irrational use. Participants with poor knowledge had 5.1 higher odds of using antibiotics irrationally than those with good knowledge (aOR=5.1, 95% CI; 1.7-15.15, P value=0.003). This is similar to the results of several other studies in Ethiopia, China, Vietnam, Malta and worldwide [7, 8, 16, 27, 28]. Participants with poor knowledge think that it is good not to finish the antibiotic course and to request the same antibiotics that they, or a friend, have used to treat similar symptoms or diseases previously. They also believe that antibiotics can be used to treat non-bacterial infections such as those causing coughs or flu, acute diarrhea, malaria, headaches or body aches. A multi-country WHO survey shows that the level of knowledge increases in more developed countries and decreases in developing countries, and that this can be linked to broader issues concerning accessibility of healthcare and medicines, and lower levels of education [4]. Education of patients concerning the rational use of antibiotics is one of the roles of dispensers in pharmacies and ADDOs. However, it appears that they are not taking the time to do this. In this study 35.5% of antibiotics without prescription were dispensed on request of the individual, with the dispenser verbally prescribing 51% of the antibiotics. Outlet type did not influence rational use of antibiotics in this study. For pharmacies 87.3% of antibiotics provided were irrational, the figure for ADDOs was 90.1%. This did not represent a significant difference between the two outlet types (P value=0.554). Similar results have been reported for ADDOs and part II shops, in that there was no significant difference in irrational antibiotic dispensing between the two outlet types [21]. Gender, health insurance status, age, marital status and employment status showed no significant association with irrational use of antibiotics in this study. This may be due to a small sample size. In other studies, gender (females), younger age, being employed and not having health insurance, were associated with the irrational use of antibiotics [26, 28, 29]. Although insignificant in this study, participants without health insurance had 1.5 higher odds of using antibiotics irrationally compared to those with insurance (aOR=1.5; 95% CI; 0.4- 5.04, P value= 0.517). In this study, level of education was not linked to knowledge of antibiotic use and the rational use of antibiotics. This contrary to theories advanced by the WHO, and may be due to the fact that people who are more educated can more readily afford antibiotics.

**Limitations**: the irrational use of antibiotics may have been underestimated because it was not possible to assess some forms of irrational use in this study. For example, participants were not followed up to observe how they actually used the antibiotics.

**Recommendations**: antibiotic resistance is a global crisis. However, the consequences of irrational use are felt more profoundly in certain areas. In the interests of the health of Tanzanians, we should reduce the use of irrational antibiotics, while ensuring they are readily accessible when indicated. The accessibility of antibiotics in drug outlets without prescription should be strictly controlled by improving stewardship and surveillance. Improved antibiotic stewardship in drug outlets is a crucial issue to be addressed. Dispensers should be well educated and informed concerning the consequences of dispensing antibiotics in treatment of upper respiratory tract symptoms, acute diarrhea and other presentations where they are unnecessary. The economic incentives that encourage overuse in the community should be eliminated. The supply chain of antibiotics should also be strictly controlled and monitored. Community education campaigns have been successful in several countries [5]. Clear messages about how to use antibiotics appropriately are required, with a strong emphasis on the fact that antibiotics play no role in the treatment of most URTS and acute diarrhea, since they are typically caused by viruses. National surveillance to monitor the susceptibility and resistance to antibiotics should be in place, with the results disseminated to policymakers and the community in order to gain financial and political support for improved antibiotic usage.

## Conclusion

Irrational use of antibiotics is highly prevalent in this population. Non-prescription use of antibiotics is the most prevalent form. Poor knowledge about antibiotic use plays a significant role in irrational antibiotic use. Surprisingly, the level of education and health insurance status were not associated with the irrational use of antibiotics. There is a need to review the accessibility of antibiotics in drug outlets.

### What is known about this topic

- Irrational use of antibiotics in African region is largely in the form of non-prescription buying and selling of antibiotics;
- Upper Respiratory Tract Symptoms are the most common reason for requesting antibiotics in the drug outlets.

### What this study adds

- Together with non-prescription usage, other forms of irrational use of antibiotics including purchasing an incomplete dosage and using antibiotics to treat non-bacterial illness account for 90% of antibiotics dispensed in the community;
- At an individual level, knowledge about the use of antibiotics influences their rational use. The individual's educational level, health-insurance status and the outlet type (pharmacy or Accredited Drug Dispensing Outlets) does not have a significant influence on the irrational use of antibiotics;
- Penicillins are not the only group of antibiotics that are commonly used irrationally in the community. Macrolides and Fluoroquinolones are also easily accessible and used irrationally.

## Competing interests

The authors declare no competing interests.
